# Plasma endothelin-1-related peptides as the prognostic biomarkers for heart failure: A PRISMA-compliant meta-analysis

**DOI:** 10.1097/MD.0000000000009342

**Published:** 2017-12-15

**Authors:** Cheng-Lin Zhang, Shang Xie, Xue Qiao, Yuan-Ming An, Yan Zhang, Li Li, Xiao-Bin Guo, Fu-Chun Zhang, Li-Ling Wu

**Affiliations:** a Department of Physiology and Pathophysiology, Key Laboratory of Molecular Cardiovascular Science, Beijing Key Laboratory of Cardiovascular Receptors Research, Ministry of Education; b Department of Oral and Maxillofacial Surgery, Peking University School and Hospital of Stomatology; c Department of Geriatrics, Peking University Third Hospital, Beijing, China.

**Keywords:** endothelin-1, heart failure, meta-analysis, prognostic factor

## Abstract

**Background:**

Most studies reported that high plasma endothelin-1 (ET-1), big ET-1, and C-terminal proET-1 (CT-proET-1) were correlated with poor prognosis of heart failure (HF). However, available evidence remains controversial. To help solve the debate, we collected all the available studies and performed a meta-analysis.

**Methods:**

We searched the databases covering Embase, PubMed, Ovid, and Web of Science on June 28, 2017. The hazard ratio (HR) or risk ratio (RR) and its 95% confidence intervals (CIs) were collected and calculated by use of a random-effect model. Heterogeneity was assessed by Cochran's Q test, and publication bias was assessed by funnel plots with Egger's and Begg's linear regression test.

**Results:**

Thirty-two studies with 18,497 patients were included in the analysis. Results showed that circulating ET-1, big ET-1, and CT-proET-1 were positively correlated with high risk of adverse outcomes, with pooled RRs (95% CIs) of 2.22 (1.82–2.71, *P* < .001), 2.47 (1.93–3.17, *P* < .001), and 2.27 (1.57–3.29, *P* < .001), respectively. In the subgroup of death as primary outcome, the pooled RRs (95% CIs) were 2.13 (1.68–2.70, *P* < .001), 2.55 (1.82–3.57, *P* < .001), and 2.02 (1.39–2.92, *P* < .001) for ET-1, big ET-1, and CT-proET-1, respectively. No significant publication bias was observed in this study.

**Conclusion:**

Our meta-analysis provided evidence that increased plasma levels of ET-1, big ET-1, and CT-proET-1 were associated with poor prognosis or mortality for HF populations.

## 1 Introduction

Heart failure (HF) is the major cause of mortality in patients with cardiovascular diseases. Over 30 million people suffered from HF globally, and the cost for HF in USA is over 30 billion US dollars each year.^[1]^ Early identification of HF patients with higher risk can lead to earlier intervention, which may potentially improve outcomes.^[2]^ Studies that investigate prognostic biomarkers for HF have been bursting over the last decades; however, short- and long-term prediction of outcomes are still challenging. Previous studies have revealed several biomarkers with prognostic value for HF, such as B-type natriuretic peptide (BNP),^[3]^ N-terminal proBNP (NT-proBNP),^[3]^ mid-regional proatrial natriuretic peptide,^[4]^ high sensitivity C-reactive protein,^[3]^ and endothelin-1 (ET-1).^[5]^ Among them, only BNP and NT-proBNP have been recommended by current guidelines.^[6]^

ET family includes 3 isoforms, namely ET-1, ET-2, and ET-3, which are encoded by 3 different genes.^[7]^ ET-1, a 21 amino acid peptide, is predominantly generated by endothelial cells and cleared by binding to its receptors in the pulmonary vascular bed.^[8]^ ET-1 is described as the most potent endogenous vasoconstrictor discovered to date.^[9]^ ET-2 and ET-3 are mainly involved in neonatal growth, intestinal functions, and central nervous system. ^[7]^ The initial product of the human ET-1 gene consists of 212 amino acid peptides, which is called prepro-ET-1. After removal of a short secretory signal sequence, prepro-ET-1 is converted into pro-ET-1. Pro-ET-1 is then cleaved by furin to generate a biologically inactive 38 amino acid precursor namedbig ET-1, and a 44 amino acid peptide named C-terminal pro-ET-1 (CT-proET-1). Mature and active ET-1 is finally excised from big ET-1 by the action of endothelin-converting enzyme.^[7]^ Plasma level of ET-1 can be altered by stimuli such as hypoxia, shear stress, lipoproteins, free radicals, and endotoxin.^[7]^ In 1995, Tsutamoto et al^[10]^ indicated that HF patients with higher level of ET-1 had elevated risk of cardiac death. Since then, many studies have investigated the predictive role of ET-1 related peptides in both acute HF (AHF) and chronic HF (CHF). Even though most of the studies have reported the relationship between higher ET-1 related peptides and clinical adverse outcomes of HF patients,^[5,11,12]^ there were still several studies holding the opposite opinion.^[13,14]^ To address this issue, we performed a meta-analysis to assess the values of ET-1, big ET-1, and CT-proET-1 in predicting prognosis in HF patients.

## 2 Methods

The study was performed according to Meta-analysis of Observational Studies in Epidemiology.^[15]^

### 2.1 Search strategy

Two investigators (Zhang and Xie) independently carried out a comprehensive literature search for original articles up until June 28, 2017. Both Medical Subject Heading terms and free text words were used to acquire relevant studies by searching databases covering Embase, PubMed, Ovid, and Web of Science. The terms searched were: “heart failure,” “endothelin,” “endothelins,” “endothelin-1,” “ET-1,” “ET,” “edn1,” “big endothelin-1,” “big ET-1,” “C-terminal proendothelin-1,” “CT-proET-1,” “prognosis”, “prognostic,” “predict,” “prediction,” “outcome,” “mortality,” and “death”. The search was limited in English language articles. Retrieved articles were screened according to the titles and abstracts, and irrelevant papers were dropped out. References of the related articles were screened to find missed papers by literature retrieval. All relevant papers were then assessed according to the inclusion and exclusion criteria as described below.

### 2.2 Inclusion and exclusion criteria

The inclusion criteria were set as follows: the included patients were diagnosed with CHF or AHF; plasma ET-1, big ET-1, or CT-proET-1 were detected in recruited participants; the endpoints were death, heart transplant, or other adverse outcomes; only prospective studies were included; results were evaluated by survival curve, HR or risk ratio (RR) with its 95% confidence interval (CI); when 2 publications reported data from overlapping samples, the study containing the larger dataset was included. Reviews, case reports, and meta-analyses were excluded. For studies without enough data to obtain RRs, the corresponding authors were contacted by email.

### 2.3 Outcomes

The endpoint evaluated in this study was adverse outcomes, which included death (all cause death, sudden death, and cardiac death) and advanced therapies (heart transplantation and HF hospitalization).^[16]^

### 2.4 Data extraction

All the data were extracted independently by 3 authors (Cheng-Lin Zhang, Shang Xie, and Xue Qiao). We extracted information from each study with the following criteria: first author, publication year, research design, country of origin, number of cases, HF type, percentage of patients in New York Heart Association (NYHA) class III-IV, follow-up period, peptides assayed, assessing methods, and cutoff value. If there was any disagreement among the 3 investigators, it was resolved by discussion with other authors until consensus was reached among them.

### 2.5 Quality assessment

The Newcastle–Ottawa Scale (NOS) was performed to evaluate the methodological quality of included studies.^[17]^ A high-quality study was defined if 1 with greater than or equal to 7 points. The assessments were performed by 2 authors (Cheng-Lin Zhang and Yuan-Ming An) independently, and discrepancies were resolved by discussion with other authors until consensus was reached.

### 2.6 Statistical analysis

All the data management and analysis were performed with STATA 11.0 software (Stata Corporation, College Station, TX). If HRs, RRs, and 95% CIs were given, data were directly extracted from the original articles. For studies with Kaplan–Meier survival curves, whereas without HRs or RRs, survival curves were read and calculated by Engauge Digitizer version 4.1 according to Parmar et al^[18]^ and Tierney et al^[19]^ Then, RRs were estimated by STATA 11.0 software (Stata Corporation). RRs were used as the universal effect sizes across studies, and HRs were directly used as RRs.^[20]^ RRs and corresponding 95% CIs were transformed to their natural logarithms (lnRR, the logarithm of RR) to stabilize variance and normalize the distribution, which were subsequently converted back to linear measures for data presentation.^[21]^

Heterogeneity was assessed by Cochran's Q test, and the significance was set at *P* value less than 0.1.^[22]^ In this case, the random-effect model was used to estimate the pooled RRs. Otherwise, the pooled RRs were estimated by the fixed-effect model.^[23]^ The inconsistency index (*I*^2^) was calculated to assess the variation caused by heterogeneity. The value of *I*^2^ between 0 and 25% represents insignificant heterogeneity, 26 and 50% as low heterogeneity, 51 and 75% as moderate heterogeneity, and more than 75% as high heterogeneity.^[24]^ Subgroup analysis was performed to explore the sources of heterogeneity. Evidence of publication bias was assessed with funnel plots by Egger's and Begg's linear regression. Significance was set at *P* value less than .05.^[25]^ Sensitivity analysis was performed by excluding each study to assess its influence on the combined RR.

## 3 Results

### 3.1 Literatures retrieval and study description

**Figure 1:**
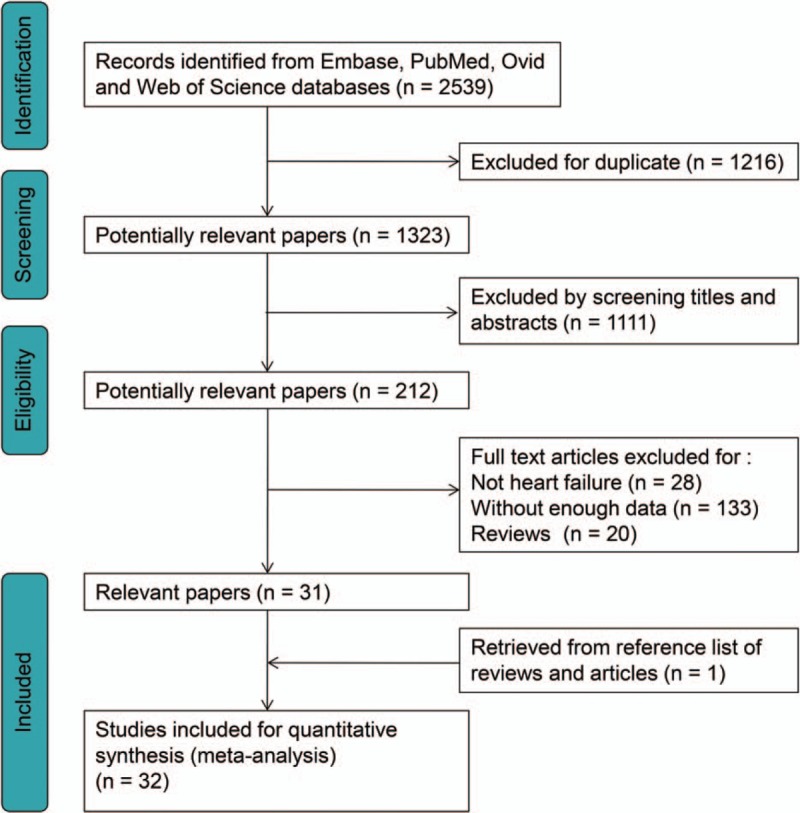
Flow diagram of literature retrieval for this study.

A total of 2539 records were identified through literatures retrieval and 1323 articles were left after excluding duplicates. Among them, 1111 records were dropped out for unconformity with our issues by reading titles and abstracts. The remaining 212 were considered potentially eligible and their full-text articles were screened. One study was retrieved from the reference.^[26]^ After serious scrutiny for eligibility, a total of 32 articles consisting of 18,497 individuals met our criteria and were included in this study (Fig. 1). Among them, 15 studies provided relevant data about ET-1 with 6151 participants,^[3,5,10,14,26–36]^ 9 studies for big ET-1 with 7928 participants,^[11,37–44]^ and 6 studies for CT-proET-1 with 4078 participants,^[45–50]^ 1 study included both ET-1 and big ET-1 with 206 participants,^[51]^ and 1 study included both ET-1 and CT-proET-1 with 134 participants.^[12]^ Diagnosis of HF was conducted according to symptom, echocardiography or New York Heart Association (NYHA) classes. Twenty-five studies were designed for CHF,^[3,10,12,14,26–29,31–39,41,42,44–47,49–51]^ 5 studies for AHF,^[3,5,30,43,48]^ 1 for dilated cardiomyopathy,^[40]^ and 1 study did not report HF classifications.^[11]^ The follow-up time ranged from 3 to 112 months, with the median time of 16 month (interquartile range from 12 to 24 months). Among the 32 studies, 3 are randomized controlled trails (RCT),^[31,37,39]^ 28 are cohort studies, and 1 study composes of both RCT and cohort study.^[50]^ 22 studies reported HR or RR, HR of 1 study was acquired from the author,^[14]^ HRs or RRs of 9 studies were estimated by methods mentioned above.^[10,29,31,32,37,38,40–42]^ HRs of 5 studies were multivariate analysis or adjusted by other confounders.^[5,26,34,44,47]^ Since 1 article only provided the subgroup data according to age of the patients, we designated the group of age less than 72 as “M. Metra (2015)^a^”, and the group of age ≥72 as “M. Metra (2015)^b^”.^[5]^

### 3.2 Quality assessment

The NOS (range 0–9 points) was performed to evaluate the quality of included studies.^[17]^ In our meta-analysis, 26 studies have quality scores of 7 to 8, others with quality scores of 6. Results of quality assessment were shown in Table 1.

**Table 1:**
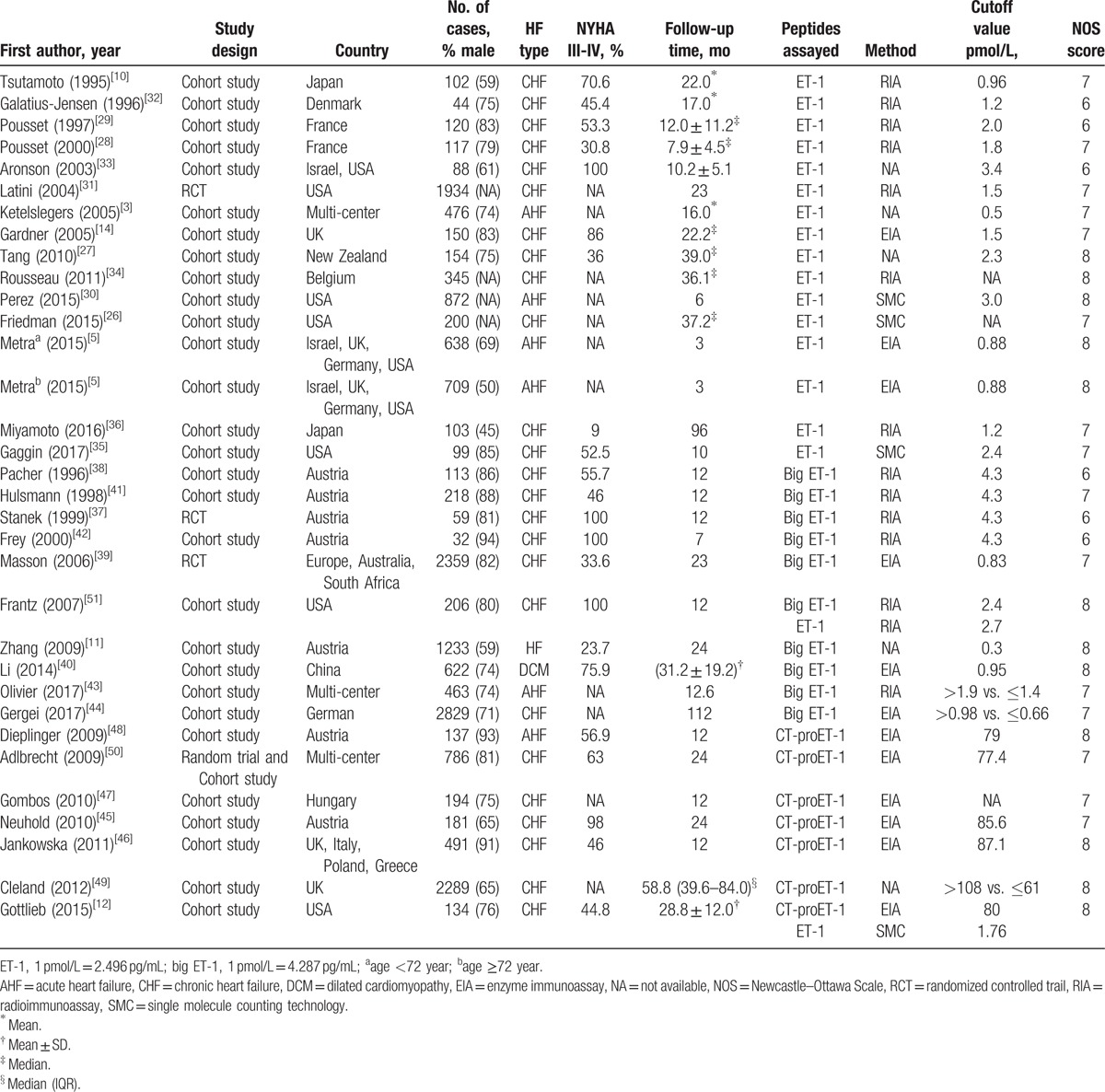
Characteristics of the studies included in the meta-analysis.

### 3.3 Circulating ET-1 is correlated with adverse outcomes and death in HF patients

**Figure 2:**
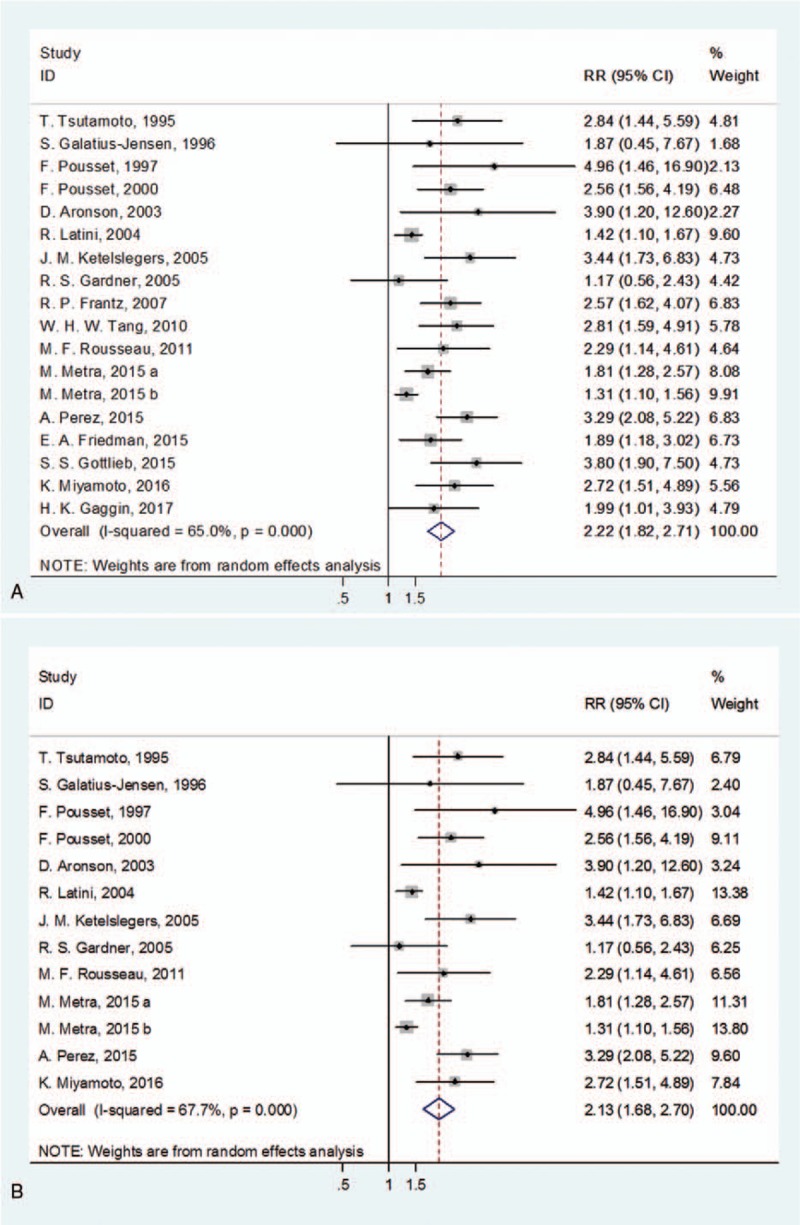
Forest plots evaluating the prognostic value of ET-1 for adverse outcomes (A) or death (B). CI = confidence intervals, RR = risk ratio; a, age <72 year; b, age ≥72 year.

In this part, 17 studies related to ET-1 were included. Moderate heterogeneity was seen cross all included studies (*I*^2^ = 65.0%, *P*_[Q-test]_ < .001). Thus, random-effect model was used to combine the HRs or RRs and 95% CIs. Results showed that high plasma level of ET-1 was significantly correlated with adverse outcomes in patients with HF (RR = 2.22, 95% CI 1.82–2.71; *P* < .001; Fig. 2A). Three methods were involved in the measurement of ET-1. Heterogeneities were reduced after subgroup analysis by detecting methods (*I*^2^ = 30.0% for enzyme immunoassay [EIA], *I*^2^ = 55.8% for radioimmunoassay [RIA], *I*^2^ = 33.9% for single molecule counting technology), indicating that the difference between methods may partly contribute to the heterogeneity for ET-1. After subgroup analysis by HF type, the pooled RRs for CHF and AHF were 2.27 (95% CI 1.82–2.82) and 2.14 (95% CI 1.34–3.44), respectively. For studies concerning severe HF (more than 80% patients were in NYHA class III-IV), the pooled RR was 2.14 (95% CI 1.15–3.96). The combined RRs were significant in follow-up time more than or equal to 24 months (RR = 2.51, 95% CI 1.93–3.26; *P* < .001), less than 12 months (RR = 2.12, 95% CI 1.47–3.05; *P* < .001), or in between (RR = 2.17, 95% CI 1.48–3.17; *P* < .001). For the 16 cohort studies, the pooled RR was 2.34 (95% CI 1.88–2.92; *P* < .001). In the 12 studies that regarded death as primary outcome, the combined RR was 2.13 (95% CI 1.68–2.70; *P* < .001; Fig. 2B). Detailed subgroup analysis results were shown in Table 2.

**Table 2:**
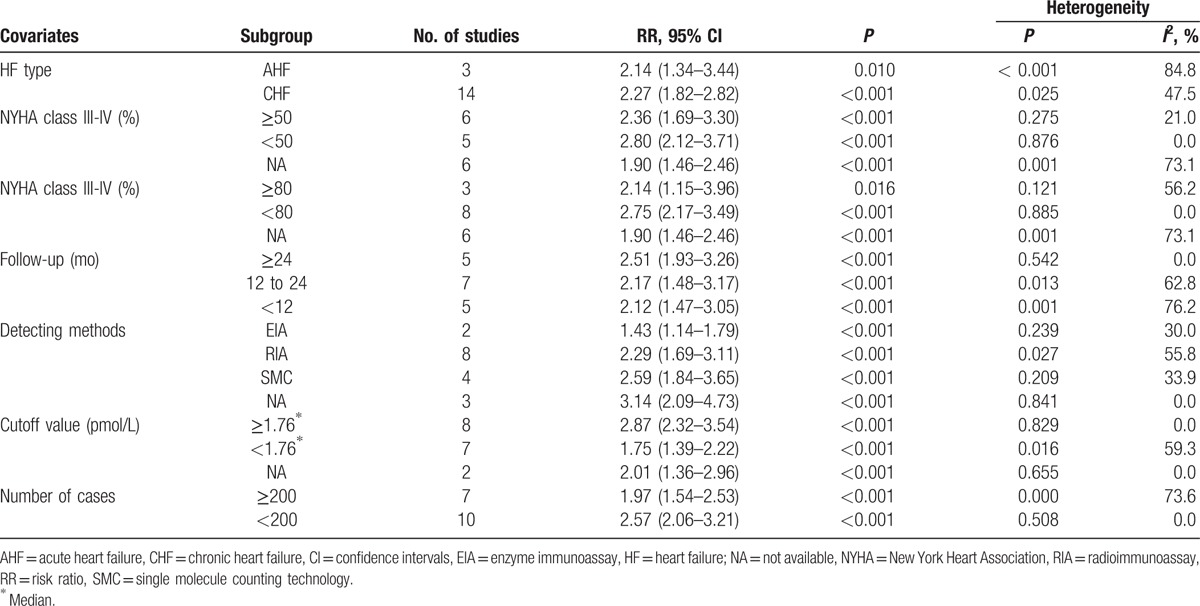
Subgroup analyses of ET-1 for adverse outcomes.

### 3.4 Circulating big ET-1 is correlated with adverse outcomes and death in HF patients

**Figure 3:**
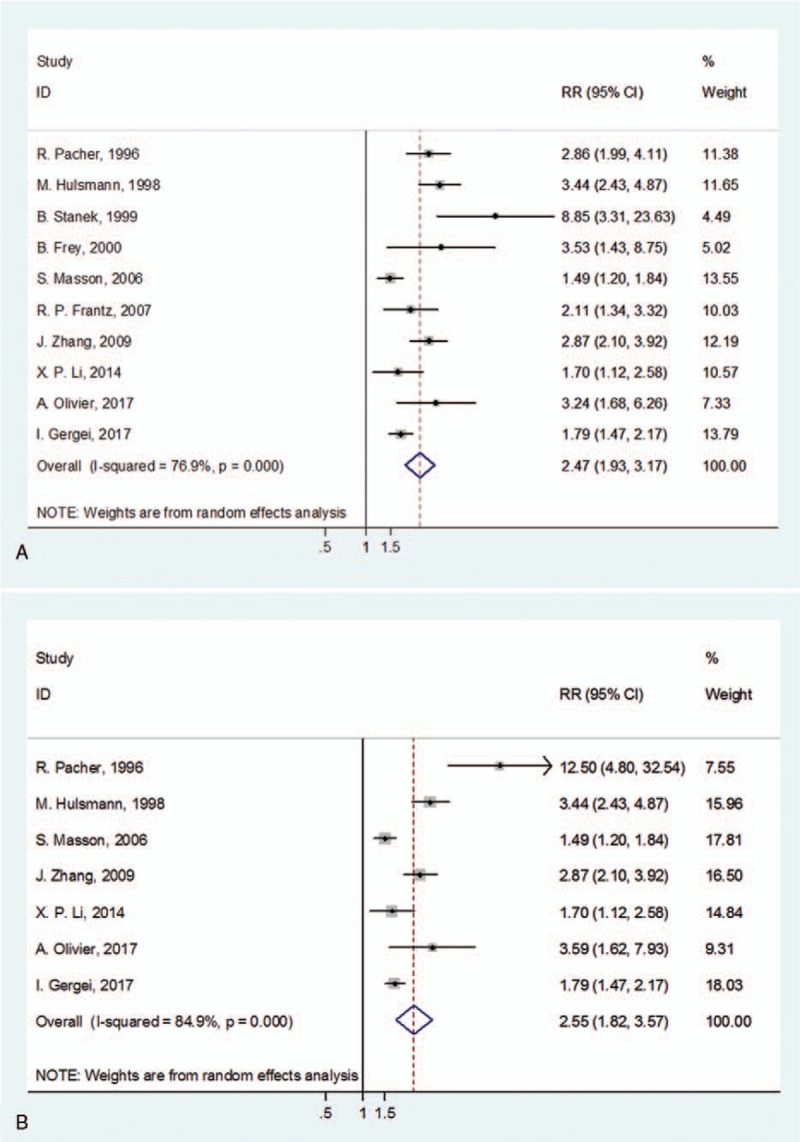
Forest plots evaluating the prognostic value of big ET-1 for adverse outcomes (A) or death (B). CI = confidence intervals, RR = risk ratio.

Big ET-1 is the biologically inactive precursor of ET-1. We further detected whether big ET-1 was valuable in predicting poor prognosis in HF patients. Considerable heterogeneity existed among the 10 available studies (*I*^2^ = 76.9%, *P*_[Q-test]_ < .001). Random-effect model was applied to combine the HRs or RRs and 95% CIs. Results showed that high level of big ET-1 was capable of indicating the adverse outcomes of HF (RR = 2.47, 95% CI 1.93–3.17; *P* < .001; Fig. 3A). Two methods were involved in the measurement of big ET-1. Heterogeneities were reduced and became acceptable in both subgroups (*I*^2^ = 0.0% for EIA, *I*^2^ = 35.6% for RIA). In the subgroup of studies that included only CHF patients, the pooled RR was 2.36 (95% CI 1.78–3.13). In the 3 studies that concerns severe HF (more than 80% patients were in NYHA class III-IV), the combined RR for adverse outcomes was 3.70 (95% CI 1.62–8.49). In the subgroup analysis of follow-up time, the prognostic effect of big ET-1 was significant in follow-up more than or equal to 24 months (RR = 2.06, 95% CI 1.49–2.85; *P* < .001) or between 12 and 24 months (RR = 2.79, 95% CI 1.84–4.23; *P* < .001). Among the 8 non-RCT cohort studies, the pooled RR was 2.48 (95% CI 1.98–3.11, *P* < .001). In the 7 studies with death as primary outcome, the pooled RR was 2.55 (95% CI 1.82–3.57; Fig. 3B). A detailed subgroup analysis results were shown in Table 3.

**Table 3:**
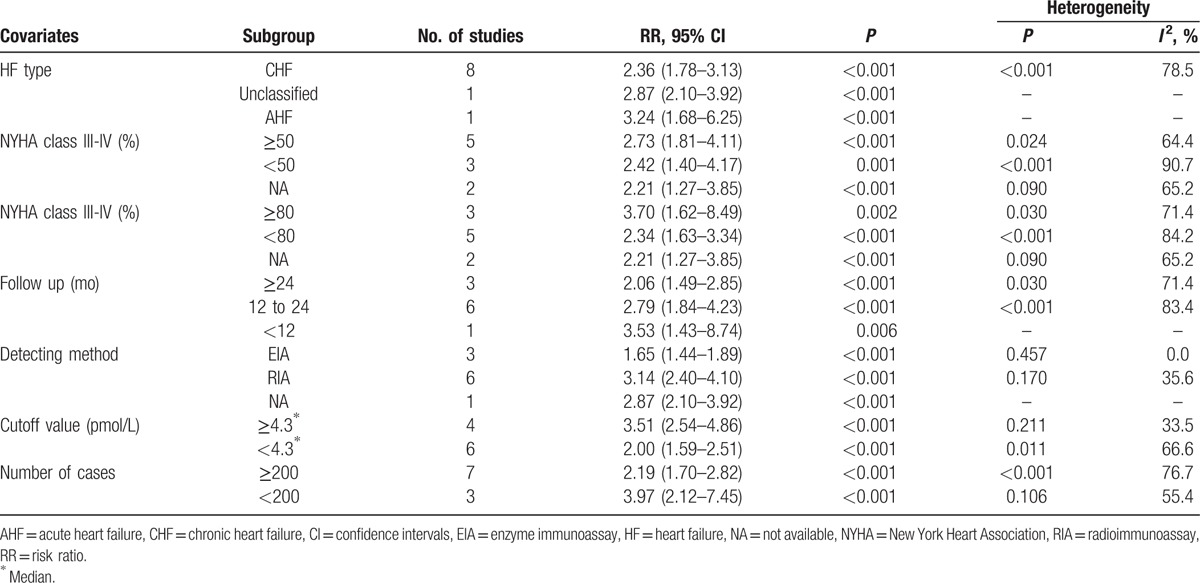
Subgroup analyses of big ET-1 for adverse outcomes.

### 3.5 Circulating CT-proET-1 is correlated with adverse outcomes and death in HF patients

**Figure 4:**
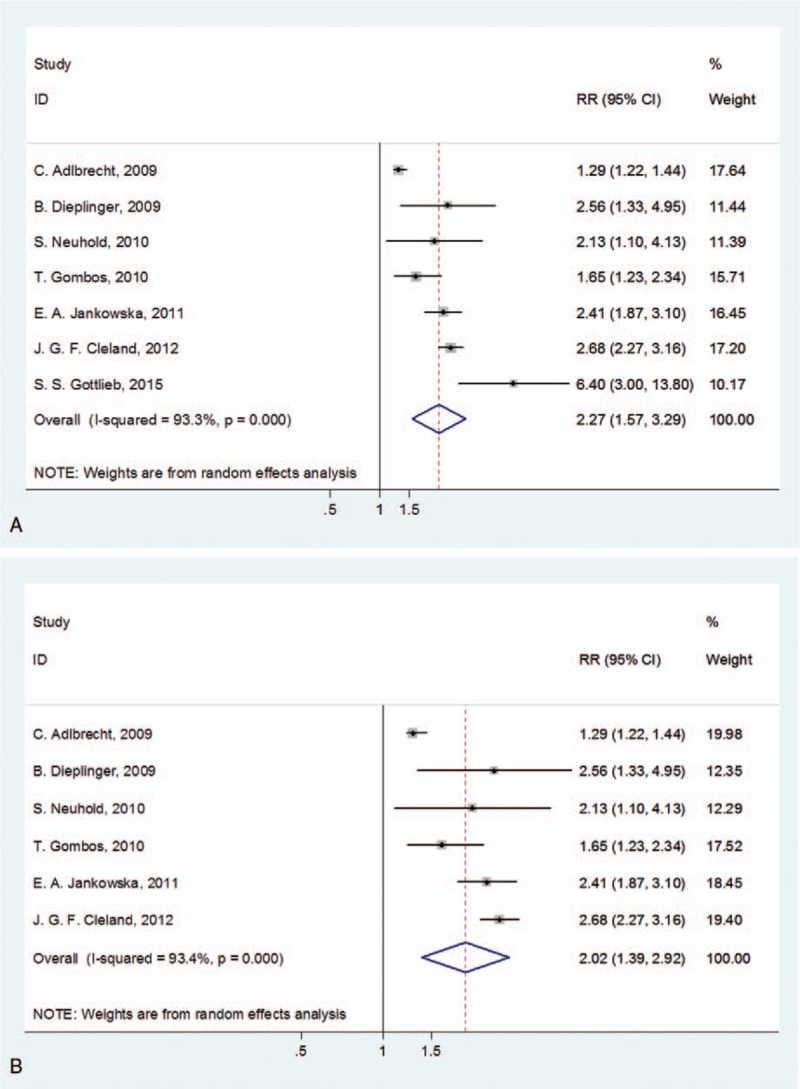
Forest plots evaluating the prognostic value of CT-proET-1 for adverse outcomes (A) or death (B). CI = confidence intervals, RR = risk ratio.

CT-proET-1 is a stable ET-1 precursor metabolite. We further detected whether CT-proET-1 was valuable for the poor prognosis of HF. Among the 7 relevant studies, a significant heterogeneity was found (*I*^2^ = 93.3%, *P*_[Q-test]_ < .001). Random-effect model was performed to combine the HRs or RRs and 95% CIs. Results showed that CT-proET-1 was also correlated with adverse outcomes of HF (RR = 2.27, 95% CI 1.57–3.29; *P* < .001; Fig. 4A). Combining the 6 studies that involved with only CHF yielded an RR of 2.24 (95% CI 1.50–3.34). In the subgroup analysis of follow-up period, the combined RRs of CT-proET-1 were 2.44 (95% CI 1.37–4.33) for longer than or equal to 24 months and 2.11 (95% CI 1.59–2.80) for 12 to 24 months. In the 6 studies with death as primary outcome, the pooled RR was 2.02 (95% CI 1.39–2.92; Fig. 4B). Detailed subgroup analysis results were shown in Table 4.

**Table 4:**
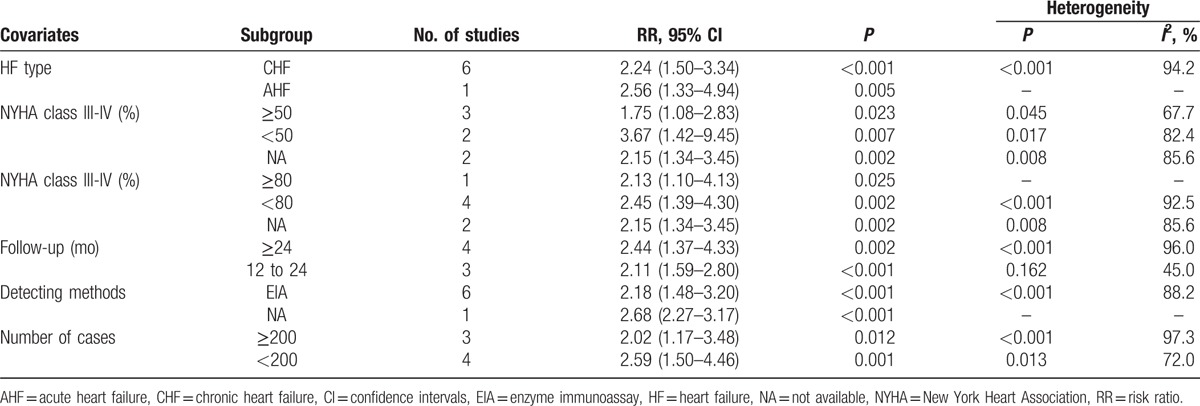
Subgroup analyses of CT-proET-1 for adverse outcomes.

### 3.6 Publication bias and sensitive analyses

**Figure 5:**
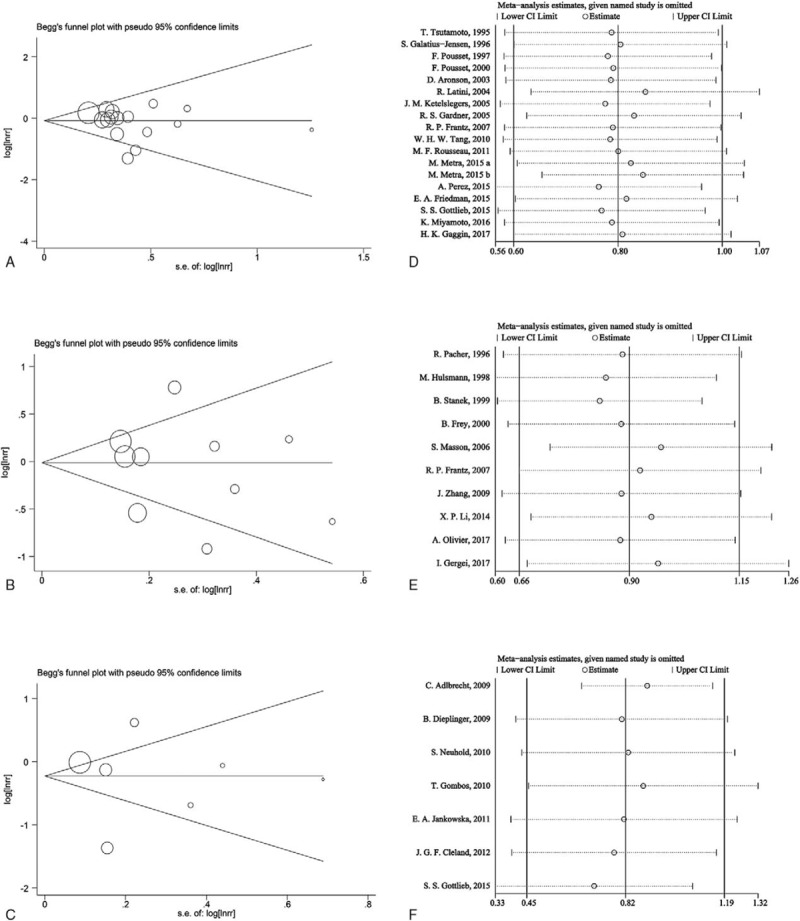
Funnel plots for ET-1 (A), big ET-1 (B), and CT-proET-1 (C); Sensitivity analyses for ET-1 (D), big ET-1 (E), and CT-proET-1 (F). ET-1 = endothelin-1.

Publication bias was assessed by performing funnel plot. No significant publication bias was found in the analyses of ET-1 (Egger's test *P* = .294; Begg's test *P* = .224), big ET-1 (Egger's test *P* = .597; Begg's test *P* = .592), and CT-proET-1 (Egger's test *P* = .813; Begg's test *P* = .548). Sensitivity analyses results demonstrated that all the estimates were changed between the lower CIs limits and the upper CIs limits, suggesting that the RR estimated were not significantly influenced by excluding each article successively (Fig. 5).

## 4 Discussion

In this study, we performed a meta-analysis and systematically reviewed the prognostic value of ET-1-related peptides in HF. Our results indicated that high circulating ET-1, big-ET-1, and CT-proET-1 were correlated with increased risk of death or adverse outcomes in HF populations.

Apart from endothelium, ET-1 is also produced by smooth muscle cells, cardiomyocytes, and cardiac fibroblasts.^[7]^ Circulating ET-1 is increased in pulmonary arterial hypertension, systematic hypertension, aging, diabetes, and myocardial infarction.^[7,52]^ This meta-analysis showed that high plasma level of ET-1 was significantly correlated with mortality or adverse outcomes in patients with HF. In the subgroup analysis, the pooled RR of ET-1 is significant in both AHF and CHF. The pooled RR of ET-1 is also significant for studies with follow-up time more than or equal to 24 months, less than 12 months, or in between. This suggests that ET-1 may have a wide range of application value in predicting outcomes of HF. Plasma ET-1 is increased with severities of HF according to the NYHA functional class.^[10,27,29]^ Notably, the heterogeneity of ET-1 was obviously reduced after subgroup analysis by the percentage of patients in NYHA class III-IV, suggesting that the severity of HF may affect the prognostic value of ET-1.

However, if it is to be a biomarker, 2 intrinsic deficiencies of ET-1 should not be ignored, that is its low plasma concentration and short half life. The average or median concentration of ET-1 in the included studies ranges from 0.5 to 3.4 pmol/L (Table 1), which requests high sensitive detecting methods to measure it. Moreover, circulating ET-1 can be quickly cleared by endothelium cells in pulmonary vascular, resulting in its half life less than 1 minute.^[8]^ These characters of ET-1 determine a higher risk of deviations between the measured ET-1 concentration in vitro and the real concentration in vivo. Therefore, it is preferable to find an alternative molecule, which can reflect the content of ET-1 more stably.

Big ET-1 is the inactive precursor of ET-1. The half life of big ET-1 in vivo is much longer than ET-1, which is about 23 minutes.^[53]^ This character may makes big ET-1 more advantageous than ET-1 to be a prognostic biomarker. However, the amino sequences between ET-1 and big ET-1 are highly similar, which makes it easier to cross-react with each other for unspecific antibodies. Meanwhile, plasma concentration of big ET-1 is very low (Table 1), which also requests a sensitive detecting method. Beneden et al^[54]^ reported that RIA was more precise than EIA in measuring big ET-1 in severe congestive HF. Consistently, our study suggests that studies using RIA tended to have higher pooled RR than studies using EIA (Table 3). Meanwhile, the heterogeneity of big ET-1 was reduced obviously after subgroup analysis by detecting method. These characters of big ET-1 may also greatly reduce its application prospects to be an independent prognostic biomarker for HF.

CT-proET-1 is derived from proET-1 and in equal molar amounts to ET-1. In contrast to ET-1 and big ET-1, circulating CT-proET-1 level is higher (∼80 pmol/L) and less cross-react with other ET-1 peptides.^[12,50]^ CT-proET-1 is stable and more resistant to rapid turnover both in vivo and in vitro, which makes it easier to obtain reliable results.^[50]^ BNP and NT-proBNP are regarded as the pivotal markers for the diagnosis and prognosis of HF. Therefore, we summarized studies that both assayed CT-proET-1 and BNP-related peptides, allowing head-to-head comparison of the prognostic accuracy of these peptides. Notably, among the 5 studies involved,^[12,45,46,48,50]^ 4 of them showed that the RR of CT-proET-1 was more robust.^[12,45,48,50]^ Therefore, it may be more prospective to use plasma CT-proET-1 as a potential prognostic biomarker in HF. However, only limited studies were carried out to this area, and more clinical trials are required to certify this hypothesis.

Background diseases and therapies should be considered when using ET-1-related peptides as prognostic markers. Plasma ET-1 concentration is correlate with clinical and hemodynamic severity under pathological conditions.^[55]^ Big ET-1 is mainly cleared in liver and kidney, as a consequence, plasma level of big ET-1 can be influenced by hepatic or renal dysfunction.^[53]^ Plasma CT-proET-1 level is correlated with renal function, age, left atrial size, and diastolic blood pressure.^[56]^ Whether an adjustment of these factors should be made in the determination of the prognostic value of ET-1-related peptides still needs further exploration.

There are several limitations in this study. First, the criteria of HF, background diseases and therapies were different among the included studies. These might be important sources of clinical heterogeneity of this study. Meanwhile, the quality assessment of RCTs by NOS scale may also cause some bias. Second, the primary outcome in our study was adverse outcomes, which might be varied among the studies. Third, 5 of 32 studies reported only with the multivariate or adjusted HR,^[5,26,34,44,47]^ which might also cause some bias for the prognostic value of ET-1-related peptides.

In summary, there is a close relationship between circulating ET-1-related peptides and the prognosis HF patients. Our study provided the meta-analysis evidence for the prognostic value of ET-1, big ET-1, and CT-proET-1 in predicting mortality or adverse outcomes in HF patients.

## References

[1] Braunwald E The war against heart failure: the Lancet lecture. Lancet 2015;385:812–24. 25467564 10.1016/S0140-6736(14)61889-4

[2] Cao TH Quinn PA Sandhu JK Identification of novel biomarkers in plasma for prediction of treatment response in patients with heart failure. Lancet 2015;385 (suppl 1):S26. 10.1016/S0140-6736(15)60341-5 26312848

[3] Ketelslegers J Zannad F Vincent J Effect of neurohormones, cytokines, and collagen markers on the risk of all-cause mortality: results from the EPHESUS trial. J Cardiac Fail 2005;11:117.

[4] Hu Z Han Z Huang Y Diagnostic power of the mid-regional pro-atrial natriuretic peptide for heart failure patients with dyspnea: a meta-analysis. Clin Biochem 2012;45:1634–9. 22981931 10.1016/j.clinbiochem.2012.08.028

[5] Metra M Cotter G El-Khorazaty J Acute heart failure in the elderly: differences in clinical characteristics, outcomes, and prognostic factors in the VERITAS study. J Card Fail 2015;21:179–88. 25573829 10.1016/j.cardfail.2014.12.012

[6] McMurray JJ Adamopoulos S Anker SD ESC guidelines for the diagnosis and treatment of acute and chronic heart failure 2012: the task force for the diagnosis and treatment of acute and chronic heart failure 2012 of the European society of cardiology. Developed in collaboration with the heart failure association (HFA) of the ESC. Eur Heart J 2012;33:1787–847. 22611136 10.1093/eurheartj/ehs104

[7] Agapitov AV Haynes WG Role of endothelin in cardiovascular disease. J Renin Angiotensin Aldosterone Syst 2002;3:1–5. 11984741 10.3317/jraas.2002.001

[8] Sirvio ML Metsarinne K Saijonmaa O Tissue distribution and half-life of 125I-endothelin in the rat: importance of pulmonary clearance. Biochem Biophys Res Commun 1990;167:1191–5. 2182027 10.1016/0006-291x(90)90649-8

[9] Kawanabe Y Nauli SM Endothelin. Cell Mol Life Sci 2011;68:195–203. 20848158 10.1007/s00018-010-0518-0 PMC3141212

[10] Tsutamoto T Hisanaga T Fukai D Prognostic value of plasma soluble intercellular adhesion molecule-1 and endothelin-1 concentration in patients with chronic congestive heart failure. Am J Cardiol 1995;76:803–8. 7572659 10.1016/s0002-9149(99)80231-8

[11] Zhang J Goode KM Cleland JGF Prognostic values of big endothelin and NT-proBNP in predicting of mortality in patients with heart failure. Eur Heart J 2009;30 (suppl 1):130.18676968

[12] Gottlieb SS Harris K Todd J Prognostic significance of active and modified forms of endothelin 1 in patients with heart failure with reduced ejection fraction. Clin Biochem 2015;48:292–6. 25541019 10.1016/j.clinbiochem.2014.12.012 PMC4363211

[13] Milo-Cotter O Cotter-Davison B Lombardi C Neurohormonal activation in acute heart failure: results from VERITAS. Cardiology 2011;119:96–105. 21912122 10.1159/000330409

[14] Gardner RS Chong V Morton I N-terminal brain natriuretic peptide is a more powerful predictor of mortality than endothelin-1, adrenomedullin and tumour necrosis factor-alpha in patients referred for consideration of cardiac transplantation. Eur J Heart Fail 2005;7:253–60. 15701475 10.1016/j.ejheart.2004.06.002

[15] Stroup DF Berlin JA Morton SC Meta-analysis of observational studies in epidemiology: a proposal for reporting. JAMA 2000;283:2008–12. 10789670 10.1001/jama.283.15.2008

[16] Gyongyosi M Wojakowski W Lemarchand P Meta-Analysis of Cell-based CaRdiac stUdiEs (ACCRUE) in patients with acute myocardial infarction based on individual patient data. Circ Res 2015;116:1346–60. 25700037 10.1161/CIRCRESAHA.116.304346 PMC4509791

[17] Wells GA Shea B O’Connell D The Newcastle-Ottawa Scale (NOS) for assessing the quality of nonrandomized studies in meta-analysis. 2011;Available at: www.ohri.ca/programs/clinical_epidemiology/oxford.asp. Accessed on November 25, 2012.

[18] Parmar MK Torri V Stewart L Extracting summary statistics to perform meta-analyses of the published literature for survival endpoints. Stat Med 1998;17:2815–34. 9921604 10.1002/(sici)1097-0258(19981230)17:24<2815::aid-sim110>3.0.co;2-8

[19] Tierney JF Stewart LA Ghersi D Practical methods for incorporating summary time-to-event data into meta-analysis. Trials 2007;8:16. 17555582 10.1186/1745-6215-8-16 PMC1920534

[20] Willi C Bodenmann P Ghali WA Active smoking and the risk of type 2 diabetes: a systematic review and meta-analysis. JAMA 2007;298:2654–64. 18073361 10.1001/jama.298.22.2654

[21] Guerra F Shkoza M Scappini L Role of electrical storm as a mortality and morbidity risk factor and its clinical predictors: a meta-analysis. Europace 2014;16:347–53. 24096960 10.1093/europace/eut304

[22] Cochran WG The combination of estimates from different experiments. Biometrics 1954;10:101–29.

[23] Cao H Wang S Zhang Z Prognostic value of overexpressed p16INK4a in vulvar cancer: a meta-analysis. PLoS One 2016;11:e0152459. 27031618 10.1371/journal.pone.0152459 PMC4816296

[24] Le HH El-Khatib C Mombled M Impact of aldosterone antagonists on sudden cardiac death prevention in heart failure and post-myocardial infarction patients: a systematic review and meta-analysis of randomized controlled trials. PLoS One 2016;11:e0145958. 26891235 10.1371/journal.pone.0145958 PMC4758660

[25] Zhou Y Liu M Li J Impact of V-ets erythroblastosis virus E26 oncogene homolog 1 gene polymorphisms upon susceptibility to autoimmune diseases: A meta-analysis. Medicine (Baltimore) 2015;94:e923. 26039128 10.1097/MD.0000000000000923 PMC4616355

[26] Friedman EA Egolum UO Shisler1 DC Elevated plasma interleukin-6, endothelin-1, and matrix metalloproteinase-9 in heart failure patients are associated with the need for advanced therapies and mortality. J Card Fail 2015;21:S90.

[27] Tang WH Shrestha K Martin MG Clinical significance of endogenous vasoactive neurohormones in chronic systolic heart failure. J Card Fail 2010;16:635–40. 20670842 10.1016/j.cardfail.2010.03.011

[28] Pousset F Masson F Chavirovskaia O Plasma adrenomedullin, a new independent predictor of prognosis in patients with chronic heart failure. Eur Heart J 2000;21:1009–14. 10901513 10.1053/euhj.1999.1904

[29] Pousset F Isnard R Trochu JN Prognostic value of plasma endothelin-1 in patients with chronic heart failure. Eur Heart J 1997;18:254–8. 9043842 10.1093/oxfordjournals.eurheartj.a015228

[30] Perez A Grodin J Hernandez A Plasma endothelin-1 independently predicts 180-day mortality in acute heart failure: an ASCEND-HF sub-study. J Am Coll Cardiol 2015;65 (suppl 10):A775.

[31] Latini R Masson S Anand I The comparative prognostic value of plasma neurohormones at baseline in patients with heart failure enrolled in Val-HeFT. Eur Heart J 2004;25:292–9. 14984917 10.1016/j.ehj.2003.10.030

[32] Galatius-Jensen S Wroblewski H Emmeluth C Plasma endothelin in congestive heart failure: a predictor of cardiac death? J Card Fail 1996;2:71–6. 8798108 10.1016/s1071-9164(96)80025-x

[33] Aronson D Burger AJ Neurohormonal prediction of mortality following admission for decompensated heart failure. Am J Cardiol 2003;91:245–8. 12521645 10.1016/s0002-9149(02)03119-3

[34] Rousseau MF Jong P Ahn SA Selective profiling of cardiac neurohormones predicts modes of death in congestive heart failure. J Am Coll Cardiol 2011;57:E1231.

[35] Gaggin HK Truong QA Gandhi PU Systematic evaluation of endothelin 1 measurement relative to traditional and modern biomarkers for clinical assessment and prognosis in patients with chronic systolic heart failure: serial measurement and multimarker testing. Am J Clin Pathol 2017;147:461–72. 28398455 10.1093/ajcp/aqx014

[36] Miyamoto K Takeuchi D Inai K Prognostic value of multiple biomarkers for cardiovascular mortality in adult congenital heart disease: comparisons of single-/two-ventricle physiology, and systemic morphologically right/left ventricles. Heart Vessels 2016;31:1834–47. 26857388 10.1007/s00380-016-0807-0

[37] Stanek B Frey B Berger R Value of sequential big endothelin plasma concentrations to predict rapid worsening of chronic heart failure. Transplant Proc 1999;31:155–7. 10083055 10.1016/s0041-1345(98)02072-7

[38] Pacher R Stanek B Hulsmann M Prognostic impact of big endothelin-1 plasma concentrations compared with invasive hemodynamic evaluation in severe heart failure. J Am Coll Cardiol 1996;27:633–41. 8606275 10.1016/0735-1097(95)00520-x

[39] Masson S Latini R Anand IS The prognostic value of big endothelin-1 in more than 2,300 patients with heart failure enrolled in the valsartan heart failure trial (Val-HeFT). J Card Fail 2006;12:375–80. 16762801 10.1016/j.cardfail.2006.02.013

[40] Li X Chen C Gan F Plasma NT pro-BNP, hs-CRP and big-ET levels at admission as prognostic markers of survival in hospitalized patients with dilated cardiomyopathy: a single-center cohort study. BMC Cardiovasc Disord 2014;14:67–75. 24885051 10.1186/1471-2261-14-67 PMC4041639

[41] Hulsmann M Stanek B Frey B Value of cardiopulmonary exercise testing and big endothelin plasma levels to predict short-term prognosis of patients with chronic heart failure. J Am Coll Cardiol 1998;32:1695–700. 9822098 10.1016/s0735-1097(98)00437-9

[42] Frey B Pacher R Locker G Prognostic value of hemodynamic vs big endothelin measurements during long-term IV therapy in advanced heart failure patients. Chest 2000;117:1713–9. 10858407 10.1378/chest.117.6.1713

[43] Olivier A Girerd N Michel JB Combined baseline and one-month changes in big endothelin-1 and brain natriuretic peptide plasma concentrations predict clinical outcomes in patients with left ventricular dysfunction after acute myocardial infarction: Insights from the Eplerenone Post-Acute Myocardial Infarction Heart Failure Efficacy and Survival Study (EPHESUS) study. Int J Cardiol 2017;241:344–50. 28284500 10.1016/j.ijcard.2017.02.018

[44] Gergei I Krämer BK Scharnagl H Propeptide big-endothelin, N-terminal-pro brain natriuretic peptide and mortality. The Ludwigshafen risk and cardiovascular health (LURIC) study. Biomarkers 2017;22:315–20. 27788598 10.1080/1354750X.2016.1252969

[45] Neuhold S Huelsmann M Strunk G Prognostic value of emerging neurohormones in chronic heart failure during optimization of heart failure-specific therapy. Clin Chem 2010;56:121–6. 19884490 10.1373/clinchem.2009.125856

[46] Jankowska EA Filippatos GS von Haehling S Identification of chronic heart failure patients with a high 12-month mortality risk using biomarkers including plasma C-terminal pro-endothelin-1. PLoS One 2011;6:e14506. 21264211 10.1371/journal.pone.0014506 PMC3022013

[47] Gombos T Forhecz Z Pozsonyi Z Adrenomedullin and endothelin-1 are novel prognostic markers in chronic heart failure. Eur J Heart Fail 2010;9:132–3.

[48] Dieplinger B Gegenhuber A Struck J Chromogranin A and C-terminal endothelin-1 precursor fragment add independent prognostic information to amino-terminal proBNP in patients with acute destabilized heart failure. Clinica Chimica Acta 2009;400:91–6. 10.1016/j.cca.2008.10.012 19000665

[49] Cleland JGF Zhang J Pellicori P Prognostic significance of plasma concentrations of C-terminal ET-1 precursor fragment in patients with suspected chronic heart failure. Eur Heart J 2012;33 (suppl 1):1095.22199121

[50] Adlbrecht C Huelsmann M Strunk G Prognostic value of plasma midregional pro-adrenomedullin and C-terminal-pro-endothelin-1 in chronic heart failure outpatients. Eur J Heart Fail 2009;11:361–6. 19190023 10.1093/eurjhf/hfp004

[51] Frantz RP Lowes BD Grayburn PA Baseline and serial neurohormones in patients with congestive heart failure treated with and without bucindolol: results of the neurohumoral substudy of the Beta-Blocker Evaluation of Survival Study (BEST). J Card Fail 2007;13:437–44. 17675057 10.1016/j.cardfail.2007.03.007

[52] Kissel CK Anderson TJ Role of endothelin-1 and endothelial dysfunction in prehypertension. Can J Cardiol 2012;28:251–3. 22284587 10.1016/j.cjca.2011.12.008

[53] Hemsen A Ahlborg G Ottosson-Seeberger A Metabolism of big endothelin-1 (1-38) and (22-38) in the human circulation in relation to production of endothelin-1 (1-21). Regul Pept 1995;55:287–97. 7761628 10.1016/0167-0115(94)00119-i

[54] van Beneden R Gurne O Selvais PL Superiority of big endothelin-1 and endothelin-1 over natriuretic peptides in predicting survival in severe congestive heart failure: a 7-year follow-up study. J Card Fail 2004;10:490–5. 15599839 10.1016/j.cardfail.2004.04.001

[55] Wei CM Lerman A Rodeheffer RJ Endothelin in human congestive heart failure. Circulation 1994;89:1580–6. 8149524 10.1161/01.cir.89.4.1580

[56] Bhandari S Davies JE Struck J Plasma C-terminal proEndothelin-1 (CTproET-1) is affected by age, renal function, left atrial size and diastolic blood pressure in healthy subjects. Peptides 2014;52:53–7. 24333656 10.1016/j.peptides.2013.12.001

